# Evaluation of Clay-Functionalized Wafers and Films for Nicotine Replacement Therapy via Buccal Mucosa

**DOI:** 10.3390/pharmaceutics11030104

**Published:** 2019-03-01

**Authors:** Joshua Boateng, Obinna Okeke

**Affiliations:** School of Science, Faculty of Engineering and Science, University of Greenwich at Medway, Central Avenue, Chatham Maritime, Kent ME4 4TB, UK; okekeobinnac@gmail.com

**Keywords:** buccal delivery, cell viability, films, HPMC, nicotine, permeation, sodium alginate, wafers

## Abstract

The functional physicochemical properties of nicotine (NIC)-loaded composite freeze-dried wafers and solvent-evaporated films comprising hydroxypropylmethylcellulose (HPMC) and sodium alginate (SA), stabilized with magnesium aluminium silicate (MAS), have been reported. The formulations were characterized for swelling capacity, mucoadhesion, in vitro drug dissolution properties in simulated saliva (SS) and PBS at pH 6.8, and ex vivo and in vitro permeation using pig buccal mucosa membrane and EpiOral^TM^ buccal tissue culture, respectively; finally, the cell viability of the EpiOral^TM^ tissues after contact with the NIC-loaded formulations was investigated using 3-(4,5-dimethylthiazol-2-yl)-2,5-diphenyltetrazolium bromide (MTT) assay and the functional characteristics compared with those of commercially available NIC strips. Swelling and NIC release from the HPMC–SA wafers were more prolonged (30 min) compared to the commercially available NIC strips which disintegrated rapidly and released the drug within 5 min. Generally, swelling, mucoadhesion, and drug release was faster in PBS than in SS, and the presence of MAS was essential for maintaining a high dose recovery compared to non-MAS formulations and commercial NIC strips, which showed lower percentage of NIC content, possibly due to evaporation during analysis. Permeation studies showed that the NIC released was able to cross both porcine buccal membrane and the EpiOral^TM^ buccal tissue, with the latter showing higher permeation flux for all the formulations tested. All the NIC-loaded, MAS-stabilized formulations showed high tissue viability, with values above 80%, showing their great potential for use as buccal delivery platforms for NIC replacement therapy to aid smoking cessation.

## 1. Introduction

Life threatening diseases such as lung cancer, chronic obstructive pulmonary disease, and cardiovascular diseases (e.g., coronary heart disease and stroke) are usually associated with cigarette smoking (U.S. Department of Health and Human Services, 2014). However, these risks are significantly decreased when smoking is curtailed, depending on factors such as age, sex, physiology, and smoking frequency. For example, the life expectancy of a smoker after cessation at 35 years could increase by 20–24% in men and 17–22% in women. However, the life expectancy of a smoker after cessation at 65 years could increase by just 2–3% in men and 4–5% in women. It is therefore more beneficial to quit smoking as early as possible [1]. An active smoker of tobacco draws in smoke known as mainstream smoke which comprises 8% tar and 92% gaseous components, while the side-stream released at the burning tail of a lit cigarette contains a higher proportion of poisonous components such as nitric oxide and carbon monoxide [2], leading to risks of severe heart disease in non-smokers [3]. 

Over the years, there have been various initiatives aimed at smoking cessation due to the health risks of tobacco, including the tobacco-free initiative (TFI) approved by the WHO. However, sudden cessation can lead to several withdrawal symptoms, including irritability, sleeplessness, and continuous cigarette craving [4]. The withdrawal symptoms can last between 2 and 12 h [5,6] and have been managed using nicotine (NIC) replacement therapies (NRTs). NIC cannot be developed as an oral pill due to its susceptibility to first-pass metabolism in the liver [7], and efforts have been made to develop alternative drug delivery systems [8] for NRT. These include chewing gums, lozenges, mouth sprays, nasal sprays, transdermal patches (e.g., Nicorette), and oral films such as NiQuitin^®^ strips [9]. Though these can achieve successful quitting of smoking, they have their limitations, and therefore novel delivery systems are required. The transdermal patch provides slow and sustained NIC release; however, it does not match the fast delivery of NIC from cigarette smoking [10]. Itching, oedema, and erythema have also been associated with transdermal patches [11]. NIC chewing gum can sometimes result in slow onset and prolonged plasma NIC levels which cannot be matched with the rapid pharmacological effect and high maximum arterial NIC levels required for relief [12]. Oral sprays allow rapid NIC absorption, but they require constant administration, and hence the bioavailability at the therapeutic level is not sustained. NRT strips such as NiQuitin^®^ deals with the challenge of chewing gum for people with dental issues or who wear dentures. However, they still face the limitation of lower absorption of NIC due to the effect of eventual swallowing [13]. Electronic NIC delivery systems (ENDS) can be misused as a substitute for cigarettes, encouraging significant numbers of smokers not to quit smoking altogether [14]. Furthermore, a recent study has demonstrated the presence of low concentrations of free radicals in e-cigarettes, which can possibly cause harm to human cells [15]. Lermer and co-workers also demonstrated the presence of potential cytotoxic metals (such as copper) and oxidants (e.g., perhydroxyl radical (HO_2_·)) normally associated with conventional cigarette, in e-cigarettes [16].

In comparison to transdermal routes, the buccal mucosa demonstrates better permeability since human buccal mucosa is composed mostly of non-keratinized cells [17,18]. The ready permeability of NIC across the buccal mucosa has been attributed to its high solubility in both water and organic solvents (LogP of 1.17) and its low molecular weight (162.2 g/mol) [19,20]. The permeability of NIC species depends on pH; however, all species of NIC can readily permeate mucosal membranes with higher permeation for un-ionized species than ionized species [21]. Porcine buccal mucosa has similar morphology and permeation to the human buccal mucosa in terms of non-keratinized cells and enzymatic activities [17], as reported for drugs such as naratriptan [22], NIC [23], buspirone [24], omeprazole [25,26], and doxepin [18]. Other buccal mucosa models such as sheep buccal mucosa have been reported in permeation studies [27,28]. EpiOral^TM^ buccal tissue comprises typical human-derived epithelial cells developed by MatTeK (MatTek Corporation, Ashland, MA, USA) and has recently been engineered and commercialized for better controlled permeation studies due to uniform and reproducible in vivo-like morphology and growth characteristics (www.mattek.com (accessed 17 October 2016)). Several researchers have utilized EpiOral^TM^ buccal tissue as a model in permeation studies [28,29,30].

The aim of this study was to investigate the functional physicochemical characteristics of clay-stabilized NIC within composite wafers and films prepared from hydroxypropylmethylcellulose (HPMC) and sodium alginate (SA) compared with commercially available NIC-loaded strips. Further, the permeation of NIC released from the composite HPMC–SA wafers and films using porcine buccal tissue and a human equivalent EpiOral^TM^ buccal tissue as well as the tissue viability of the EpiOral^TM^ tissue after coming in contact with the NIC-loaded wafers and films was investigated using 3-(4,5-dimethylthiazol-2-yl)-2,5-diphenyltetrazolium bromide (MTT) assay.

## 2. Materials and Methods

### 2.1. Materials

Hydroxypropylmethylcellulose (HPMC; Methocel K100 Premium LV) was a gift from Colorcon Limited (Dartford, UK), and magnesium aluminium silicate (MAS) was a gift from R.T. Vanderbilt Company Inc. (Norwalk, CT, USA). Sodium hydroxide, potassium dihydrogen phosphate, and gelatine were purchased from Fluka Analytical (Buchs, Switzerland). Nicotine (liquid form), sodium alginate (SA; molecular weight 120,000–190,000 g/mol, mannuronate/guluronate ratio 1.56), submaxillary mucin from porcine stomach, PBS tablets (pH 7.4), 3-(4,5-dimethylthiazol-2-yl)-2,5-diphenyltetrazolium bromide (MTT), Krebs–Ringer bicarbonate buffer, and dimethyl sulfoxide (DMSO) were all obtained from Sigma Aldrich (Dorset, UK). Sodium acetate, trimethylamine, and glycerol (GLY) were purchased from Fisher Scientific (Loughborough, UK). Commercially available NIC-loaded strips (NiQuitin^®^) was purchased from a local pharmacy (Gillingham, Kent). Calcium chloride dehydrate, sodium chloride, sodium phosphate dibasic, magnesium chloride hexahydrate, potassium carbonate hemihydrate, sodium phosphate monobasic monohydrate, sodium acetate, and trimethylamine were all purchased from Fisher Scientific (Loughborough, UK). The EpiOral^TM^ buccal tissue kit (ORL-200) was purchased from MatTek Corporation (Ashland, MA, USA).

#### Preparation of Simulated Saliva

Simulated saliva was prepared using the formula summarized in Table 1.

### 2.2. Selected Optimized Formulations

The following formulations in Table 2 were selected for the investigation of the effect of PBS and SS (pH 6.8; ionic strength, 0.04) on swelling and in vitro drug dissolution characteristics. The MAS and non-MAS formulations were prepared as previously described, respectively [32,33].

### 2.3. Swelling and In Vitro Mucoadhesion Studies

The swelling capacities of wafers and films, as well as commercially available NIC-loaded strips, were determined by immersing each formulation into 5 mL of PBS (pH 6.8) or SS (pH 6.8; ionic strength, 0.04 M). The percentage swelling index was investigated by recording change in weight at time intervals of 2 min up to 30 min. For every time point, the medium was carefully removed to obtain an accurate weight of the sample and replaced with fresh medium. Three replicates were performed for each sample and swelling index (%) was calculated using Equation [34].

(1) $$Swelling\;index=\frac{Ws-Wd}{Wd}\times 100$$

where *W*_d_ = dry weight of polymeric wafer/film, *W*_s_ = weight of wafer/film after swelling.

Adhesion test was performed on the wafers and films using a TA. HD plus Texture Analyzer (Stable Micro Systems, Surry, UK) in tensile mode and fitted with a 5-kg load cell. Films were cut to dimensions matching the mathematical area of wafers (a circle with diameter = 15.5 mm) and attached to an adhesive probe (75 mm diameter) of the TA instrument using double-sided adhesive tape. Gelatine gel (6.67% (*w*/*v*)) was prepared by dissolving gelatine powder in water at 70 °C, poured into a Petri dish (86 mm diameter), and placed in a fridge overnight to set into a solid gel to represent the buccal mucosa surface. Mucin solution (2% *w*/*v*) was prepared by dissolving mucin powder in SS and 0.5 mL evenly spread on the surface of the set gelatine gel to simulate the buccal mucosa. The probe with film or wafer attached was lowered to make contact with the model buccal mucosa surface with an applied force of 1.0N and detached after a contact time of 60 s. Mucoadhesive strength was determined by the maximum adhesive force (*F*_max_) required to detach the sample from the model buccal surface, work of adhesion was determined by the area under the force-distance curve, while cohesiveness represents the distance the wafers/films travelled till they detached from the model buccal surface.

Finally, the swelling capacities and mucoadhesion profiles of the commercial NIC-loaded strip were compared to those of the formulated composite HPMC–SA wafers and films.

### 2.4. In Vitro Drug Dissolution Studies

In vitro drug dissolution of the wafers and films was performed with the help of a Franz-diffusion cell apparatus. The receptor compartment was filled with 8 mL of PBS or SS (pH 6.8) with a mesh on the receptor surface. The donor and receptor compartments were sealed with paraffin, to limit evaporation and held together by a pinch clamp. The system was placed on a water bath at 37 °C with magnetic stirring at approximately 200 rpm. The samples were weighed and placed on the mesh between the donor and receptor compartments. At predetermined time intervals, 0.5-mL aliquots of the dissolution media were withdrawn using a 1-mL syringe, filtered through a 0.45-µm cellulose acetate membrane, transferred into glass vials, and analysed using HPLC. The aliquots withdrawn at each time point, was replaced with fresh buffer solution, in order to maintain a constant volume of dissolution media. The percentage drug released from the formulations was calculated and plotted against time (*n* = 3). Further, the in vitro drug dissolution of the commercial strip was performed with the protocol described above but with only SS and compared to the drug release profiles of the HPMC–SA wafers and films.

### 2.5. Comparison of Release Profiles using Difference and Similarity Factors

The equations of Moore and Flanner were adopted in the calculation of the difference (*f*_1_) and the similarity (*f*_2_) factors in comparing the release profiles of the wafers and films in SS and PBS as well as between the wafers, films and commercial NIC-loaded strips. The difference factor value (*f*_1_) measures the percent error between two curves over all time points, while the similarity (*f*_2_) factors value is a logarithmic transformation of the sum-squared error of differences between the test *T*_j_ and reference products *R*_j_ over all time points [35]. The difference (*f*_1_) and the similarity (*f*_2_) factors were calculated using Equations (2) and (3) below:

(2) $$f_{1}=\;\frac{\sum_{j=1}^{n}\left|R_{j}-T_{j}\right|}{\sum_{j=1}^{n}R_{j}}\;\times 100$$

(3) f2=50×log{(1+(1n)∑j=1n|Rj−Tj|2)} ×100

The drug release profiles are considered similar if the *f*_1_ values is close to 0 and the *f*_2_ values is close to 100 or if *f*_1_ is lower than 15 and *f*_2_ value is higher than 50. According to FDA recommendations, a similarity is declared for two drug release profiles if *f*_2_ is between 50 and 100 [36,37,38].

### 2.6. Drug Content (% Loading/Recovery)

The content of NIC in commercial strip was assayed and the results were compared to that obtained for the HPMC–SA wafers and films. The content of NIC in all the samples (*n* = 3) was assayed, by accurately weighing each sample and dissolving in 10 mL of distilled water. The resulting solution was collected into a syringe, filtered through a 0.45-µm cellulose acetate membrane, transferred into sample vials, placed in the HPLC sample chamber, and analysed using HPLC conditions as described below.

#### HPLC Analysis

NIC was analysed by HPLC using an Agilent 1200 HPLC instrument (Agilent Technologies, Cheshire, UK) with an auto sampler. The stationary phase used was a C-18 reverse-phase column, 4.6 × 250 mm (Phenomenex, Cheshire, UK). Sodium acetate solution, methanol and trimethylamine, (88:12:0.5 *v*/*v*) were used as mobile phase with pH adjusted to 4.2 using glacial acetic acid, at a flow rate of 1 mL/min and UV detection at 259 nm [39]. The retention time of NIC was detected at approximately 4.5 min. A calibration curve was plotted from NIC standards ranging from 40 µg/mL to 400 µg/mL (*R*^2^ = 0.9994).

### 2.7. Permeation and Tissue Viability Studies

The HPMC–SA wafers and films were investigated for NIC permeation across porcine and EpiOral^TM^ buccal tissues. Non-treated EpiOral^TM^ tissue was used as a negative control in MTT assay for tissue integrity (viability) studies.

#### 2.7.1. Ex Vivo Buccal Permeation Studies

Ex vivo buccal permeation studies of NIC released from wafers (MAS and non-MAS wafers) and films (MAS films) were carried out using Franz diffusion cell with a diffusional surface area of 0.6 cm^2^. Fresh porcine buccal tissue was obtained from a local slaughterhouse (Kent, UK) and was immediately stored in a container containing Krebs–Ringer bicarbonate buffer (modified with sodium bicarbonate) and used within 2 h of slaughter [28,40]. The tissues were trimmed with a scalpel to a thickness of 1–3 mm and washed with physiological PBS (pH 6.8) at 37 °C. Membranes were mounted on a Franz diffusion cell between the donor and the receiver (8 mL of 0.01 M PBS; pH 6.8) compartments with the epithelial side facing the donor compartment. The receiver compartment was allowed to equilibrate at 37 °C for 30 min while stirring at 200–400 rpm. After the equilibration period, 0.5 mL of 0.01 M PBS were poured into the donor compartment and 20–30 mg of optimized wafers or films were placed in the donor compartment with the mucoadhesive layer in contact with the epithelial surface. The donor and the receiver chambers were held together tightly with a cell clamp and sealed with parafilm to limit evaporation. Samples (0.5 mL) were collected at predetermined time intervals from the port of the receiver compartment and replaced with the same amount of PBS in order to maintain a steady volume for 4 h. The collected samples were analysed using HPLC. Permeation flux (*J*) was determined using Equation (4).

(4) $$J\;=\;\frac{dQ}{dt\;}.\frac{1}{A},$$

*J* = steady state flux; *d*_Q_/*d*_t_ = amount of drug permeated; *A* = effective diffusion area.

#### 2.7.2. In Vitro Buccal Permeation Studies (EpiOral^TM^ Buccal Tissue)

EpiOral^TM^ assay medium (MatTek, Ashland, MA, USA) was pre-warmed to 37 °C for 30 min. Using a sterile technique, 0.3 mL/well of EpiOral^TM^ assay medium were pipetted into four wells of a 24-well plate and labelled as 1 h equilibration. The remaining wells were labelled as 0.5, 1, 2, 3, and 4 h. The EpiOral^TM^ samples were transferred into the 1-h equilibration wells containing the pre-warmed assay medium and placed in a 37 °C, 5% CO_2_ incubator for 1 h. After 1 h equilibration, the EpiOral^TM^ was transferred into the 0.5-h labelled well, treated with 0.5 mL donor solution (0.01 M PBS) into which 15 mg of wafers and/or film were added with the mucoadhesive layer in contact with the apical surface of the EpiOral^TM^ buccal tissue, and returned to the incubator. After the elapsed time point (0.5 h) the tissue was moved to the next time point (i.e., 1, 2, 3, and 4 h) till the total elapsed time (4 h). Then, 50 μL of the receiver fluid were collected at predetermined time intervals and transferred to a vial for HPLC analysis. Permeation flux (*J*) was determined using Equation (4).

#### 2.7.3. Permeation Correlation between Porcine and EpiOral^TM^ Buccal Tissues.

The permeability of NIC across the porcine buccal tissue and EpiOral^TM^ engineered human buccal tissue epithelium was further investigated to determine the correlation using a correlation curve of EpiOral^TM^ cumulative permeation against the porcine cumulative permeation curve of wafers and film. Linear regression (*R*^2^) obtained from the curve of film and wafers was compared.

#### 2.7.4. Tissue Viability (MTT Assay) of EpiOral^TM^ Tissues after Permeation Studies

Following the EpiOral^TM^ permeation studies, the tissue inserts used were transferred into a 24-well plate filled with MTT solution (0.3 mL) dissolved in PBS (5 mg/mL) and incubated for 3 h. After incubation, the MTT was gently extracted from all well plates and the cultures were extracted in 2 mL of DMSO for 2 h with gentle shaking (120 rpm). The aliquots (*n* = 3) of the extracts (200 μL) were placed in a 96-well plate and the absorbance of the extracted (purple-coloured) formazan was determined using a Multiskan microplate photometer at 540 nm. The viable cells had the greatest amount of MTT reduction and hence the highest absorbance values. Relative cell viability was calculated for each tissue used during permeation as a percentage of the mean negative control tissues (*n* = 3). The average percentage cell viability of optimized wafers and films was plotted using the non-treated tissues as negative control, which have 100% viability.

### 2.8. Statistical Analysis

Statistical analysis was performed using student *t*-test and/or one-way ANOVA to compare the results. The results were expressed as mean ± standard deviation and significant differences were determined at a level of *p* < 0.05.

## 3. Results

### 3.1. Swelling Studies

#### 3.1.1. HPMC/SA Wafers and Films

**Figure 1:**
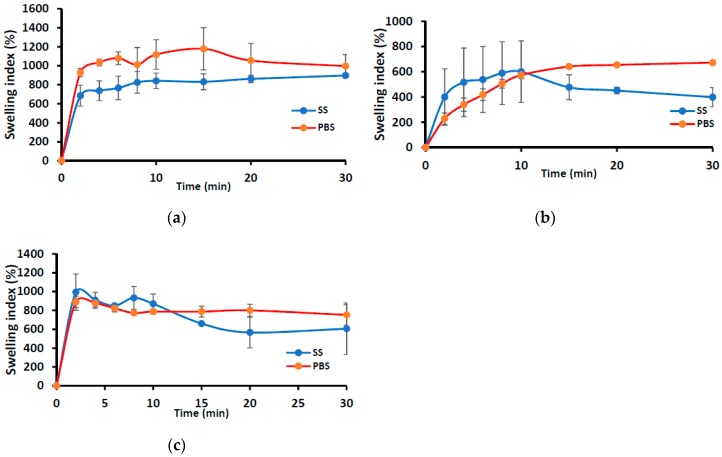
Swelling profiles (i.e., % swelling index against time) of (**a**) MAS wafer, (**b**) MAS film, and (**c**) non-MAS wafer in SS and PBS (*n* = 3, ± SD).

Figure 1 shows the swelling profiles (% swelling index against time) of the wafers (MAS wafer and non-MAS wafer) and films (MAS film) in SS and PBS media. The wafers (i.e., MAS and non-MAS wafers) in general demonstrated higher swelling index in both SS (maximum swelling index; 1178 ± 221%) and PBS (maximum swelling index; 897 ± 26%) than the films (swelling index of 600 ± 243%, 672 ± 10% in PBS and SS maximum, respectively). There was a difference in the swelling profile of all formulations (both wafers and films) between SS and PBS medium. MAS wafer showed a statistically significant difference (*p* < 0.05) between SS and PBS swelling profiles, while MAS film and non-MAS wafer showed no statistically significant difference (*p* > 0.05 and *p* > 0.05, respectively). However, the structural integrity of MAS films and non-MAS wafers was observed to decrease after 10 min.

#### 3.1.2. Swelling Profile of Commercial Strip versus Optimized Wafers and Films

**Figure 2:**
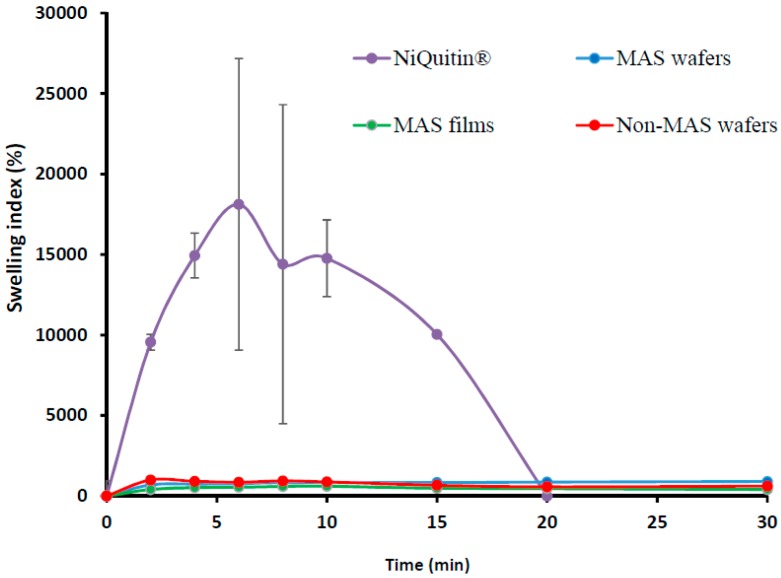
Swelling profile (i.e., % swelling index against time) (*n* = 3, ± SD) of NiQuitin^®^, HPMC–SA wafers, and films.

The swelling profile of commercial NIC-loaded strips in SS is shown in Figure 2. The swelling index demonstrated a maximum swelling index of 18118 ± 943% at 6 min, which was far higher than the wafers and films, but started to decline sharply after the maximum swelling index and hence completely eroded within 20 min.

### 3.2. In Vitro Mucoadhesion

#### 3.2.1. HPMC/SA Wafers and Films

**Figure 3:**
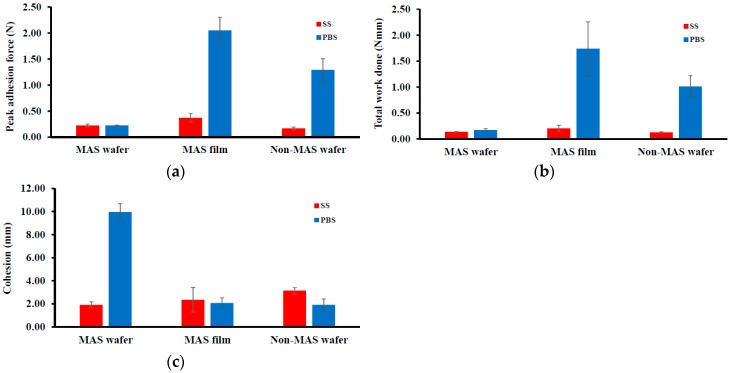
Differences in mucoadhesive profiles of optimized wafers and films (*n* = 3, ± SD) in PBS and SS: (**a**) peak adhesive force (N), (**b**) total work done (N mm), (**c**) cohesiveness (mm).

Figure 3 shows the adhesive properties (PAF, TWA, and cohesiveness) of wafers and films. The peak adhesion force (PAF) or *F*_max_ of the wafers and films were higher in PBS compared to SS. In PBS, the maximum value of 2.05 ± 0.25 N was observed in MAS film compared to MAS wafers and non-MAS wafers, with a PAF of 0.23 ± 0.003 N and 1.29 ± 0.22 N, respectively. In SS, the maximum value decreased but was also observed to be higher in MAS film (0.37 ± 0.08 N) compared to MAS wafers (0.23 ± 0.03 N) and non-MAS wafers (0.17 ± 0.03 N). TWA and cohesiveness also followed a similar trend as PAF, with a decrease in TWA and cohesiveness in SS. However, the maximum cohesiveness was observed in MAS wafers (9.96 ± 0.71 N) compared to MAS film (2.07 ± 0.45 N) and non-MAS wafers (1.92 ± 0.51 N).

#### 3.2.2. In Vitro Mucoadhesion of Commercial Strip Compared with Optimized Wafers and Films

**Figure 4:**
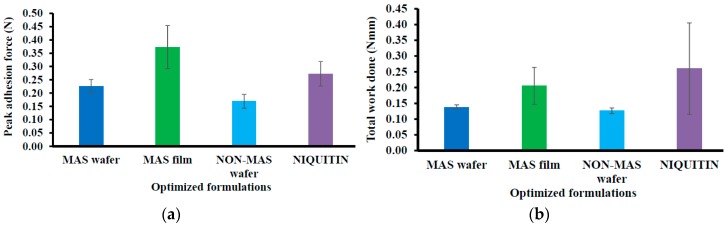

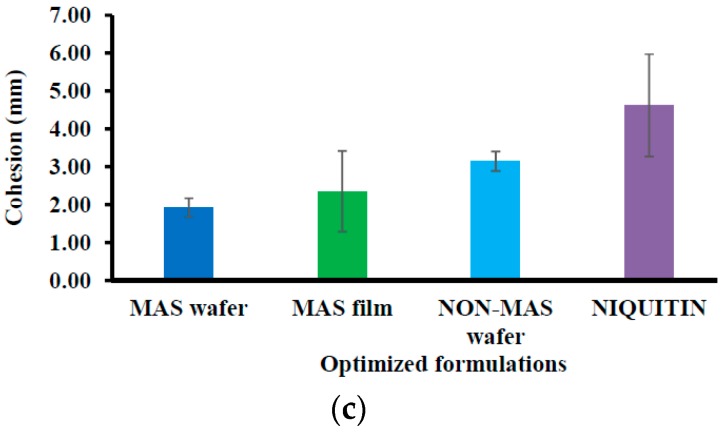
Mucoadhesive profiles of NiQuitin^®^ and HPMC–SA wafers and films (*n* = 3, ± SD) in the presence of SS: (**a**) peak adhesive force (N), (**b**) total work done (Nmm), (**c**) cohesiveness (mm).

Figure 4 demonstrated the mucoadhesion profile of NiQuitin^®^ compared to the wafers and films using SS. The highest PAF value was demonstrated in MAS films (0.37 ± 0.08 N) in comparison with NiQuitin^®^ (0.27 ± 0.05 N) and wafers (i.e., MAS and non-MAS wafers; 0.23 ± 0.03 N and 0.17 ± 0.03 N, respectively). However, NiQuitin^®^ strips showed the maximum value in TWA and cohesion with 0.26 ± 0.15 N mm and 4.62 ± 1.35 mm, respectively, compared to the optimized wafer and film formulation.

### 3.3. In Vitro Drug Dissolution

#### 3.3.1. HPMC–SA Wafers and Films

**Figure 5:**
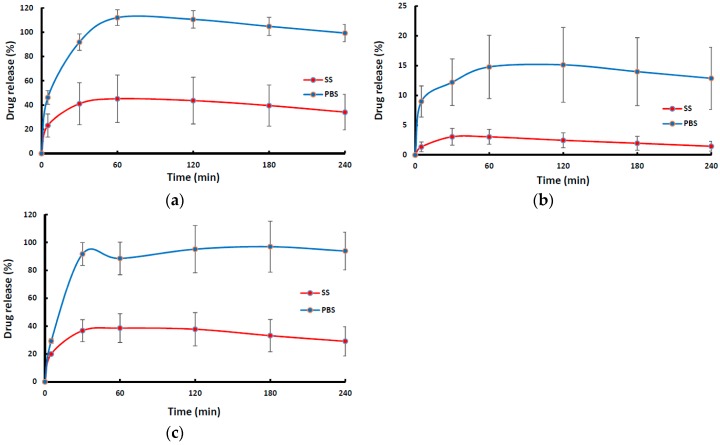
In vitro drug release profile (*n* = 3, ± SD) of NIC from formulations; (**a**) MAS wafer, (**b**) MAS film, and (**c**) non-MAS wafer, in SS and PBS.

Figure 5 shows the drug dissolution profiles of NIC-loaded wafers and films in SS and PBS. The dissolution profiles of wafers (MAS wafer and non-MAS wafer) showed a rapid drug release with about 80–100% of NIC released within 60 min in PBS, while the MAS film demonstrated a more sustained release profile as NIC was gradually released from the polymeric matrix in PBS. On the other hand, NIC release from wafers and films in the presence SS appear to demonstrate a much slower NIC release profile for all wafers and films within the first 2 h of release, though a much longer release period could have shown much more release of the drug. The NIC release from MAS wafer, MAS film, and non-MAS wafer in SS and PBS demonstrated a statistically significant difference (*p* < 0.05, *p* < 0.05 and *p* < 0.05, respectively).

#### 3.3.2. Commercial Strip (NiQuitin^®^) versus Optimized Wafers and Films in SS

**Figure 6:**
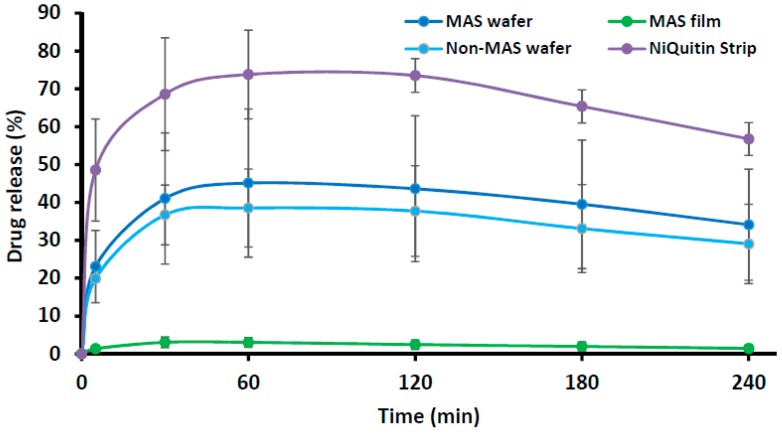
In vitro drug release profile of NiQuitin^®^ strip and HPMC/SA wafers and films (*n* = 3, ± SD).

Figure 6 compares the in vitro drug dissolution profiles of NiQuitin^®^ commercial strip and optimized formulations (i.e., MAS wafer, MAS film and non-MAS wafer) in SS. The release profile of NiQuitin^®^ showed a more rapid drug release from the polymer matrix into SS than the wafers and films. The in vitro release profiles of NiQuitin^®^ and wafers and films showed a statistically significant difference (*p* < 0.05).

### 3.4. Comparison of Drug Dissolution Profiles

The drug dissolution profiles of the formulated wafers in PBS and SS were compared using *f*_1_ and *f*_2_ values (i.e., similarity or difference, respectively) relative to a selected reference formulation, as shown in Table 3. The MAS film was chosen as a reference as it exhibited the lowest rate of drug release.

Further, the drug dissolution profiles of all the formulated wafers and films in SS were compared using NiQuitin^®^ strips as the reference formulation and the results shown in Table 4.

### 3.5. Drug Content (% Loading/Recovery) of Commercial Strips and Formulated Wafers and Films

**Figure 7:**
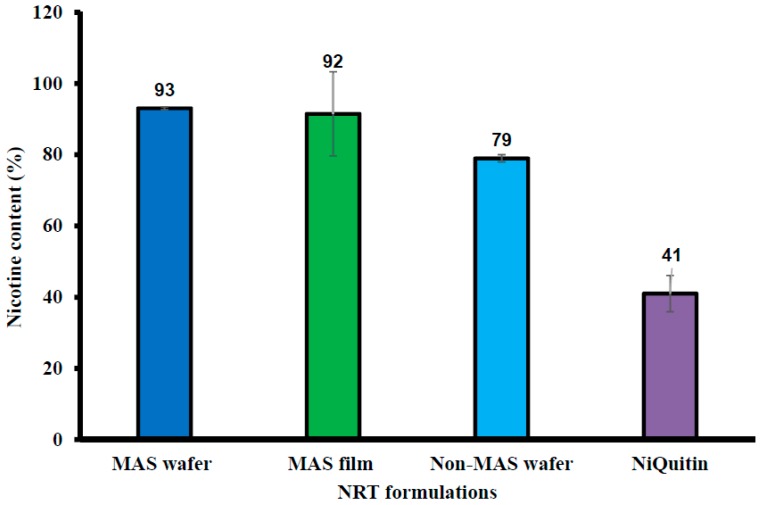
NIC content (%) in NiQuitin^®^, HPMC–SA wafers and films (*n* = 3, ± SD).

Figure 7 shows the percentage drug in NIC-loaded commercial strips, formulated wafers, and films. The commercial strips demonstrated the lowest NIC content (%), with 41 ± 5.10% NIC. MAS wafer and film showed the highest NIC content, with 93 ± 0.40% and 92 ± 11.82% of NIC, respectively. MAS was also confirmed to have a significant effect on NIC content as well as the formulation technique as non-MAS film was demonstrated to have low NIC content in [32].

### 3.6. Ex Vivo Buccal Permeation Studies (Porcine Buccal Tissue)

**Figure 8:**
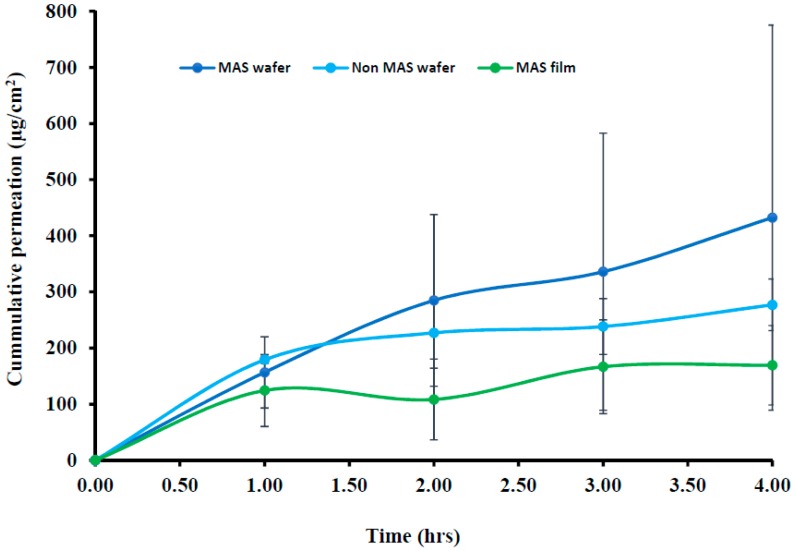
Cumulative permeation curve of wafers and films using porcine buccal tissue (*n* = 3, ± SD).

The cumulative permeation curves of formulated wafers (MAS and non-MAS wafers) and films (MAS films) using porcine buccal tissues are shown in Figure 8, and the permeation flux (*J*) of the formulations is shown in Table 5. NIC permeation in the formulated wafers and films demonstrated a high cumulative permeation above 100 μg/cm^2^ with the wafers (MAS and non-MAS wafers), in general demonstrating higher cumulative permeation than the films (MAS films).

The highest cumulative permeation and permeation flux (*J*) was shown in MAS wafers with the maximum cumulative permeation of 432.30 ± 343.04 μg/cm^2^ within 4 h and permeation flux (*J*) of 108.08 ± 85.76 μg/cm^2^/h, while the lowest cumulative permeation and permeation flux (*J*) was shown in MAS films with the maximum cumulative permeation of 169.30 ± 70.67 μg/cm^2^ within 4 h and permeation flux (*J*) of 42.33 ± 17.67 μg/cm^2^/h.

### 3.7. In Vitro Buccal Permeation Studies (EpiOral^TM^ Buccal Tissue)

**Figure 9:**
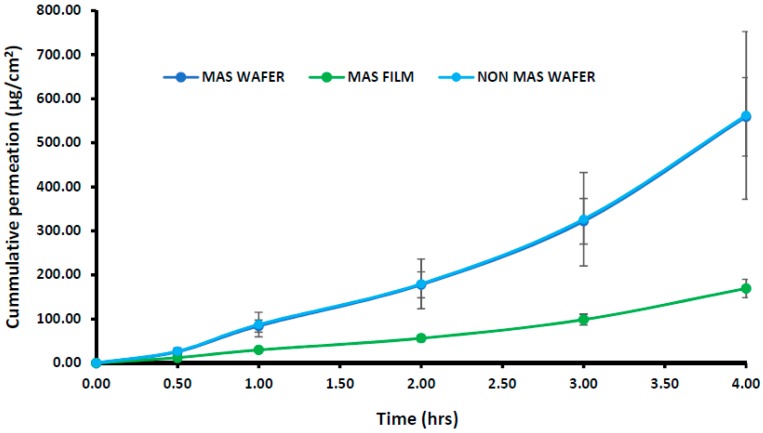
Cumulative permeation of optimized wafers and films using EpiOral^TM^ engineered buccal tissue (*n* = 3, ± SD).

The cumulative permeation curves of the NIC released from wafers and films using EpiOral^TM^ buccal tissue are shown in Figure 9. The permeation flux (*J*) of NIC from wafers and films is also shown in Table 5, with the permeation of NIC from wafers and films demonstrating a lag-time of 30 min. The highest cumulative permeation within 4 h and permeation flux (*J*) was observed for wafers with the maximum cumulative permeation of 562.22 ± 190.20 μg/cm^2^ (Non-MAS wafers) and permeation flux (*J*) of 40.55 ± 47.55 μg/cm^2^/h. The MAS films demonstrated a lower cumulative permeation and permeation flux within 4 h, with a maximum cumulative permeation of 169.234 ± 20.89 μg/cm^2^ and permeation flux (*J*) of 42.31 ± 5.22 μg/cm^2^/h. EpiOral^TM^ buccal tissues demonstrated a higher flux than porcine buccal tissues.

### 3.8. Permeation Correlation between In Vitro Porcine Buccal Tissue and EpiOral^TM^ Engineered Buccal Tissue

**Figure 10:**
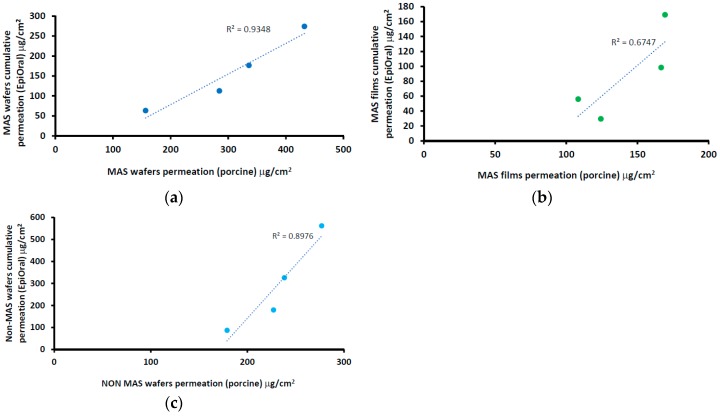
Correlation between porcine and EpiOral^TM^ cumulative permeation curve for (**a**) MAS wafers, (**b**) MAS films, and (**c**) non-MAS wafers.

The correlation between the cumulative permeation curve of NIC in wafers (i.e., MAS and non-MAS wafers) and films using a porcine buccal tissue and EpiOral^TM^ buccal engineered tissue is shown in Figure 10. The results showed a positive correlation between wafers and films, with an increase in cumulative permeation using EpiOral^TM^ buccal tissue as the values for porcine buccal tissue increased. The wafers generally showed higher regression than optimized films. MAS wafers (Figure 10a) demonstrated the highest linear regression coefficient (0.935) while MAS films (Figure 10b) demonstrated the least linear regression coefficient (0.675).

### 3.9. Tissue Viability (MTT Assay) of EpiOral^TM^ Tissues after Permeation Studies

**Figure 11:**
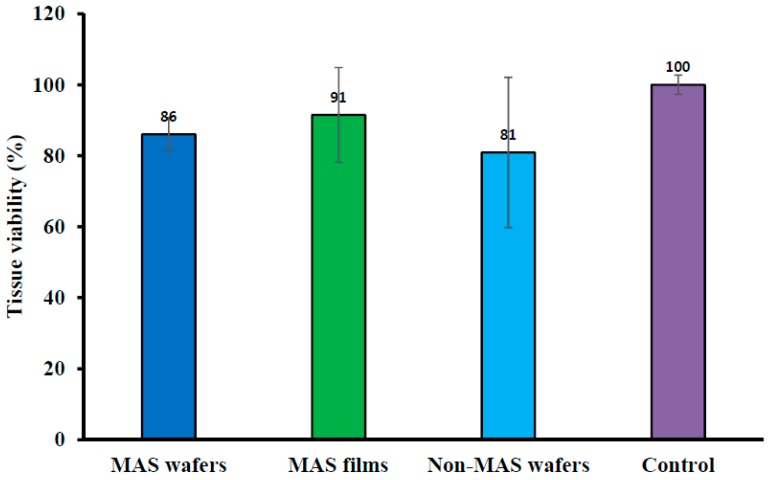
EpiOral^TM^ tissue viability after permeation studies of MAS wafers, MAS films, non-MAS wafers, and negative (non-treated) control (*n* = 3, ± SD).

Cell viability of EpiOral^TM^ engineered buccal tissue following permeation studies of wafers and films measured with MTT assay is shown in Figure 11. There was some reduction in the activity of cell enzymes (mitochondrial succinate dehydrogenase) in all optimized wafer (i.e., MAS and non-MAS wafers) and film (MAS films) formulations as there was a lower percentage of cell viability in comparison to the negative non-treated control (100% viability). However, MAS films demonstrated a high percentage cell viability (91 ± 13%) in comparison with wafers (MAS wafers; 86 ± 4% and non-MAS wafers; 81 ± 21%).

## 4. Discussion

The design of a buccal drug delivery system involves the successful application of the optimized formulation to the buccal mucosa and the absorption of the drug either rapidly or in a controlled manner over a stipulated period. The immediate microenvironment of the buccal mucosa region plays a vital role in modulating the drug release with matrix swelling via hydration in the dissolution medium, diffusion and erosion of the polymer matrix as the main mechanisms of a controlled release formulation [41,42]. Human saliva therefore plays a major role in the release mechanism of a buccal drug delivery system and it is vital in functional characterization of swelling and in vitro release studies to consider the components i.e., presence of electrolytes such as sodium, calcium, potassium, chloride, phosphate, bicarbonate, and magnesium.

The swelling results demonstrated a significantly higher swelling index in PBS than SS (*p* < 0.05) especially for MAS wafer. This implies that the presence of electrolytes and predominantly negatively charged mucin increases ionic interaction, which affected the swelling capacity of both optimized wafers and films. The diffusion of PBS into MAS wafer can be attributed to electro-osmosis i.e., generation of an electric field by mobile ions in MAS (silicate, magnesium, and aluminium) with accelerated flow, which induces high diffusivity of water molecules associated to these ions. This could also explain why MAS wafers demonstrated higher swelling capacity than non-MAS wafers. However, the presence of SS electrolytes reduced swelling capacity of the formulations by creating an ionic pressure gradient. This excess pressure was introduced with the difference in concentrations of ions in the formulation and in SS, which decreases the diffusion rate of SS into the formulations [43]. Furthermore, the ions and mucin present in SS compete for available ionic interaction with SA and MAS in the MAS wafers and films and SA in optimized non-MAS wafer, hence reducing the rate of hydration [26], and this eventually affects the rate of drug release from the swollen formulations.

The mucoadhesion of optimized wafers and films depends on various mechanisms of interaction with the mucosa surface such as adsorption, wetting, diffusion and mechanical theories [44]. The mucoadhesion in the presence of SS is slightly lower than in PBS, as previously reported [33] suggesting that the presence of higher ionic interactions by components in SS such as sodium, calcium, potassium, chloride, phosphate, bicarbonate, magnesium, and mucin could potentially interact with the negatively charged SA and MAS group. This limited the ionic interaction as well as hydrogen bonding with the mucin in the mucoadhesive model system used, because the ions and mucin present in SS compete for bonding sites on the wafer and film polymeric matrices [45]. The high mucoadhesion in the films compared to wafers could be attributed to adhesion based on liquid-to-solid affinity (wetting theory), with the film’s large surface area playing a role in adhesion compared to optimized wafers with lower total surface area and lesser contact with the mucosal surface because of the presence of sponge-like pores. NiQuitin^®^ strips also followed similar mucoadhesion profiles as MAS films, since they are essentially a sheet-like formulation. However, the ionic effect on mucoadhesion from the high concentration of charged components in SS is minimal, with only methyl acrylic acid–ethyl acrylate copolymer (anionic) in NiQuitin^®^ strips as a competing site for ionic interaction of SS components compared to MAS film, which contained both SA (anionic polymer) and MAS (amphoteric clay). In addition, the high hydration and swelling properties of methyl acrylic acid–ethyl acrylate copolymer in NiQuitin^®^ improved the diffusion properties, which encouraged chain entanglement (diffusion theory of mucoadhesion).

One of the major challenges of dealing with free NIC base is its volatility, and NIC readily evaporates in an unstable formulation. Based on the conditions used in the analysis of the formulated wafers and film formulation, the low NIC content in NiQuitin^®^ strips can be attributed to loss of NIC over time, including during analysis. This suggests that the hydrogen bonding between the copolymer and NIC was not stable enough to stabilize NIC within the commercial strips. The high NIC content in MAS formulation can be attributed to strong ionic interaction between the negatively charged silicate and the partially positively charged NIC in combination with hydrogen bonding between NIC and the composite polymers in the formulation, hence stabilizing NIC in the formulation.

The in vitro drug release from optimized wafers and films significantly depends on the hydration, which leads to swelling of the polymeric dosage form and eventual drug diffusion from the swollen matrix [42]. As described above, the presence of electrolyte and mucin in SS caused a decrease in the swelling capacity of the formulated wafers and films. These components create an ionic pressure between the high concentration of charged components such as mucin and electrolyte in the SS and the ions in the formulation. At the molecular level, this initial slow rate of release could be related to ionic interactions between the drug, charged MAS, ionized alginate, and also competition with the ions present in SS as compared to PBS. This type of effect, especially in the presence of SS, has been reported in previous studies [26], where release was significantly slower in SS than in PBS. It is possible that a longer period of release will show release of higher amounts of drug with eventual matrix erosion, and this will need to be investigated in future studies. However, for the purposes of buccal delivery, which was the main objective of the current study, the expected duration of release for effective permeation and systemic action is usually 2 h, since most of the drug will eventually end up in the saliva and be swallowed via the gastro-intestinal route.

The results of the in vitro drug release studies also demonstrated a similar trend to the swelling data with a decrease in the release profile of wafers and films formulation in SS as compared to the release profile in PBS. This implies that the presence of electrolytes and mucin slows down the release rate from the formulations over time, hence avoiding dumping of NIC in the buccal mucosa region [26].

NiQuitin^®^ strip is composed mainly of anionic copolymers i.e., methacrylic acid–ethyl acrylate copolymer, and these copolymers contribute to the fast dissolution properties of NiQuitin^®^ formulation and the rapid release of NIC upon contact with saliva. Other components of NiQuitin^®^ include triethyl citrate used as a plasticizer, peppermint flavour and sucralose for taste masking, and sodium hydrogen carbonate used as a buffer, all of which have high affinity for water. The swelling profile (% swelling index against time) observed in NiQuitin^®^ strips showed that the maximum swelling profile of NiQuitin^®^ strips was attained within 6 min of contact with the SS medium. Methyl acrylic acid–ethyl acrylate is an anionic based copolymer that responds to environmental pH. Anionic hydrogels are usually ionized at higher pH above its pK_a_ and become un-ionized below its pK_a_. The rapid dissolving process of the copolymer used in NiQuitin^®^ strips was activated with an increase in pH upon contact with the SS solution by the neutralizing base (sodium hydrogen carbonate) which then creates an osmotic swelling force in the copolymer network by the presence of hydroxyl ions [46]. The rapid ingress of SS into the polymeric matric results in the eventual rapid erosion of the polymer matrix after its optimum swelling capacity as observed in NiQuitin^®^ swelling profile (Figure 6). However, in the case of the MAS film and non-MAS wafer the swelling in SS was slower compared to the NiQuitin^®^. Furthermore, the swelling appeared sustained in SS compared to PBS with the PBS profiles showing decrease in % swelling with time, suggesting that the erosion effects on MAS film and non-MAS wafer in SS was significantly lower than in NiQuitin^®^. This could explain the significant differences observed in the eventual drug release in the first 2 h.

Compared with the HPMC–SA wafers and films, NiQuitin^®^ strips showed a rapid release of NIC in less than 30 min. This can be attributed to the rapid swelling of the strips in response to environmental pH triggered by the neutralizing base in addition to the other highly water soluble components (triethyl citrate and sucralose). However, the sharp decrease in swelling of the NiQuitin^®^ strips after the maximum swelling point at 6 min can be attributed to low concentration (8 mL of medium in receiving chamber) of sodium hydrogen carbonate which impacts on osmotic swelling force with a high ionic strength of the SS. The limitation of using the Franz diffusion cell to assess the release of NIC from formulations such as NiQuitin^®^ is that the product is designed to be placed on the tongue and then pressed by the roof of the mouth. This increases the duration of drug release relative to actual application as the pressure applied on the strip by the roof of the mouth will have increased the disintegration of the polymer matrix and hence result in higher dissolution rate compared to the experimental results obtained in this study. The Franz diffusion cell used in this project could not model the pressure applied on the NiQuitin^®^ both by the tongue and the roof of the mouth, but did demonstrate the dissolution of NiQuitin^®^ upon contact with SS. Using FDA guidelines in comparing two or more dissolution profiles (similarity (*f*_2_) and difference (*f*_1_) factors), the wafers demonstrated a difference in dissolution with NiQuitin^®^ as the MAS wafers and non-MAS wafers showed *f*_2_ similarity factor <15 and *f*_1_ difference factor <50, which could be related to the differences in micro- architecture.

This study also aimed to investigate NIC permeability when released from the wafers and films using porcine and human engineered EpiOral^TM^ buccal tissues as model buccal mucosa membranes. The toxicity of the buccal cells was assessed as the mucoadhesive formulations were loaded with NIC, which a known toxic compound at high doses [47]. The buccal route offers an ideal opportunity for NIC delivery as it bypasses the NIC degradation (such as the hepatic first-pass effect) that occurs when administered by the conventional oral route. NIC has the ability to more easily penetrate the buccal route than the skin [21,48]. The buccal permeability of the wafers and films with different physicochemical properties and attributes such as swelling and release properties was necessary, as it is essential to achieve the required bioavailability for eventual therapeutic action. Furthermore, in order to assess the reliability of the permeation results, both porcine and human engineered EpiOral^TM^ buccal tissues were utilized and compared.

The most important properties that affect the permeability of a drug compound through a tissue membrane is its lipophilicity and molecular weight [49]. Lipophilicity is usually expressed in terms of octanol–water partition coefficient (log *P*). NIC possesses a low log *P* value (1.17) and a low molecular weight of 162.23 g/mol, which make it highly permeable via the buccal mucosa at physiological pH (6.8), with un-ionized NIC permeating via the transcellular pathway, while ionized NIC permeates via the paracellular route [18]. The permeation of NIC via porcine and EpiOral^TM^ buccal tissue in this study demonstrated a high flux value (between 40 μg/cm^2^/h and 150 μg/cm^2^/h) over a time period of 4 h. Similar high flux values of NIC have been observed in previous studies using porcine esophageal mucosa as a model membrane [50] and human skin [20]. Pongjanyakul and co-workers demonstrated in their permeation studies a high permeation curve of NIC between 400 μg/cm^2^ and 800 μg/cm^2^ within 8 h and also showed a decrease in film permeation rate as the MAS ratio in film increased, which was a similar case to this study as NIC permeation decreased in the MAS films [50]. The use of porcine and EpiOral^TM^ buccal tissue in these studies demonstrated a good correlation. However, EpiOral^TM^ buccal tissue demonstrated a higher permeation flux (*J*) than porcine buccal tissue, which can be attributed to fatty tissues beneath the ex vivo porcine buccal mucosa tissue.

Hydration, swelling, and release rate of NIC from the formulations played a role on the permeation flux across the porcine and EpiOral^TM^ buccal tissues. Wafer formulations (MAS and non-MAS wafers) showed a higher swelling index, which can be attributed to their high porosity, which allows rapid ingress of dissolution medium into the polymer matrix [32]. The increased hydration and swelling of wafers played a role in the release rate of NIC from the polymer matrix which influenced the permeation flux in both porcine and EpiOral^TM^ buccal tissue models with higher flux in comparison to films. The increased NIC release from optimized wafers allows a higher concentration diffusion gradient towards absorption across the buccal membrane compared to the films. Film formulations (MAS films), on the other hand, demonstrated a lower permeation flux in both porcine and EpiOral^TM^ buccal tissue models which can be attributed to lower rates of hydration, swelling and release [32,51]. The diffusion of fluid into film formulation is relatively slow owing to the continuous polymeric sheet and absence of pores which therefore affects the hydration and swelling of the formulations and subsequent release rate of NIC from the swollen gels with low concentration diffusion gradient towards absorption across the buccal membrane. This implies that optimized wafers can provide a more rapid action while optimized films can provide a prolonged action.

MTT was utilized to assess the tissue viability of EpiOral^TM^ after contact with the optimized wafers and films. The assay investigates the reduction of yellow MTT to an insoluble purple formazan predominantly by enzymes (succinate dehydrogenase) found in the mitochondria of viable cells [52,53,54]. The MTT assay demonstrated some level of cytotoxicity by NIC, with the non-viable cell’s inability to reduce yellow MTT to purple formazan. NIC has been reported by Chang and co-workers to suppress the growth of periodontal ligament fibroblast (PDLF) as well as inhibit cell proliferation and decrease protein synthesis, with an increase in NIC concentration [47]. NIC-induced cytotoxicity was shown in the current study, with optimized wafers demonstrating 14% non-viable cells in MAS wafers and 19% non-viable cells in non-MAS wafers; however, a lower percentage of non-viable cells (8%) was shown in MAS films. The increase in NIC-induced cytotoxicity in optimized wafer formulations was as a result of increased hydration and swelling of wafers, which resulted in more rapid release of NIC from the polymeric matrix. The film formulation, however, demonstrated slow hydration and swelling, leading to slow NIC release with lower concentration diffusion gradient thereby resulting in lower numbers of non-viable cells in EpiOral^TM^ buccal tissue. However, in general, the results of cell viability (MTT) assay for both the wafers and films demonstrated that the formulations can be considered safe to use, since the percentage of viable cells was more than 70% after exposure, which is the acceptable international standard [55,56].

## 5. Conclusions

HPMC–SA based wafers and films were analysed for swelling and NIC release properties using SS as medium to demonstrate the effect of the constituents of SS such as electrolytes and mucin on formulation performance. The formulations demonstrated reduced swelling properties in SS compared to PBS, attributed to the ionic pressure created by the high concentration of ions and mucin present in SS. The drug release profile of the optimized formulations demonstrated a slow release profile within 30 min, which correlated directly with the swelling profile. On the other hand, the swelling profile of NiQuitin^®^ strips demonstrated a rapid swelling within 6 min and also a higher swelling compared to the HPMC–SA based wafers and films but eroded rapidly after reaching its optimum swelling capacity. NIC content in the wafers and films also demonstrated higher content (recovery) of NIC in comparison to NiQuitin^®^ strips, attributed to the ionic interaction between the MAS and NIC as well as hydrogen bonding of NIC to the SA within the composite formulations which stabilized the drug within the wafers and films. Furthermore, the rapid swelling rate of NiQuitin^®^ strips was directly related to its rapid release of NIC from the polymer matrix in comparison with HPMC–SA wafers and films. NIC released from the wafers demonstrated a higher permeation flux than the corresponding films for both porcine and EpiOral^TM^ buccal tissue, attributed to the differences in functional physical (hydration, swelling and release) properties of optimized wafers and films. A good correlation was achieved between NIC cumulative permeation in porcine and EpiOral^TM^ buccal tissues, with wafers (MAS wafers) showing the highest correlation coefficient. As a result of the high concentration of NIC released from the wafers, the percentage of viable cells in EpiOral^TM^ buccal tissue was reduced the most in wafers compared to the films. However, the HPMC–SA based wafers and films can be considered safe, as the percentage of viable cells was >70%.

## Figures and Tables

**Table 1:** Simulated saliva (SS) compositions used in this study [31].

Composition	Concentration (g/L)
Calcium chloride dehydrate	0.228
Sodium chloride	1.017
Sodium phosphate dibasic	0.204
Magnesium chloride hexahydrate	0.061
Potassium carbonate hemihydrate	0.603
Sodium phosphate monobasic monohydrate	0.273
Submaxillary mucin	1.00
pH	6.8

**Table 2:** Composite HPMC–SA wafers and films used for this study. NIC: nicotine; HPMC: hydroxypropylmethylcellulose; SA: sodium alginate; GLY: glycerol; MAS: magnesium aluminium silicate.

NIC-Loaded Formulations	HPMC (% *w*/*v*)	SA (% *w*/*v*)	GLY (% *w*/*v*)	MAS (% *w*/*v*)	NIC (g)
MAS wafer	1.25	0.75	-	0.25	0.20
MAS film	1.25	0.75	2.00	0.25	0.20
Non-MAS wafer	1.25	0.75	-	-	0.20

**Table 3:** Similarity and difference factors for drug release profiles of optimized wafers and films (**a**) between optimized wafers and film in SS and (**b**) between optimized wafers and film in PBS.

**(a)**		
**Optimized formulations (SS)**	**Similarity factor (*f*_2_)**	**Difference factor (*f*_1_)**
MAS wafer	22.07	1597.71
MAS film	Reference	Reference
Non-MAS wafer	25.53	1362.02
**(b)**		
**Optimized formulations (PBS)**	**Similarity factor (*f*_2_)**	**Difference factor (*f*_1_)**
MAS wafer	3.87	624.56
MAS film	Reference	Reference
Non-MAS wafer	6.82	535.57

**Table 4:** Similarity (*f*_2_) and difference factor (*f*_1_) of optimized wafers and films compared to NiQuitin^®^ strips.

Optimized Formulations	Similarity Factor (*f*_2_)	Difference Factor (*f*_1_)
MAS wafer	28.58	41.43
MAS film	10.10	96.54
Non-MAS wafer	24.68	49.56
NiQuitin^®^	Reference	Reference

**Table 5:** Permeation flux (*J*) for NIC from optimized wafers and films through porcine mucosa membrane and EpiOral^TM^ tissue construct.

	Formulation	Flux (*J*) (μg/cm^2^/h) (mean ± SD, *n* = 3)
Porcine tissue	MAS wafers	108.08 ± 85.76
	MAS films	42.33 ± 17.67
	Non-MAS wafers	69.22 ± 11.50
EpiOral^TM^ tissue	MAS wafers	139.74 ± 22.29
	MAS films	42.31 ± 5.22
	Non-MAS wafers	140.55 ± 47.55
